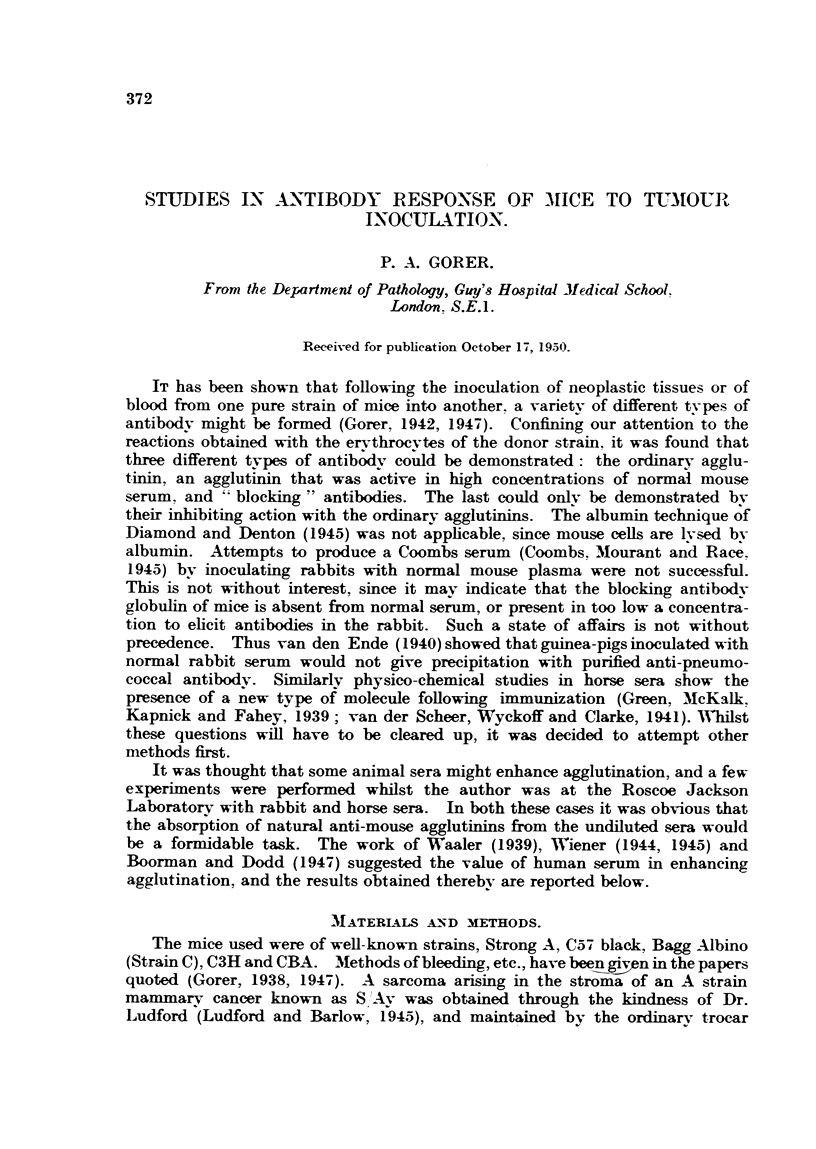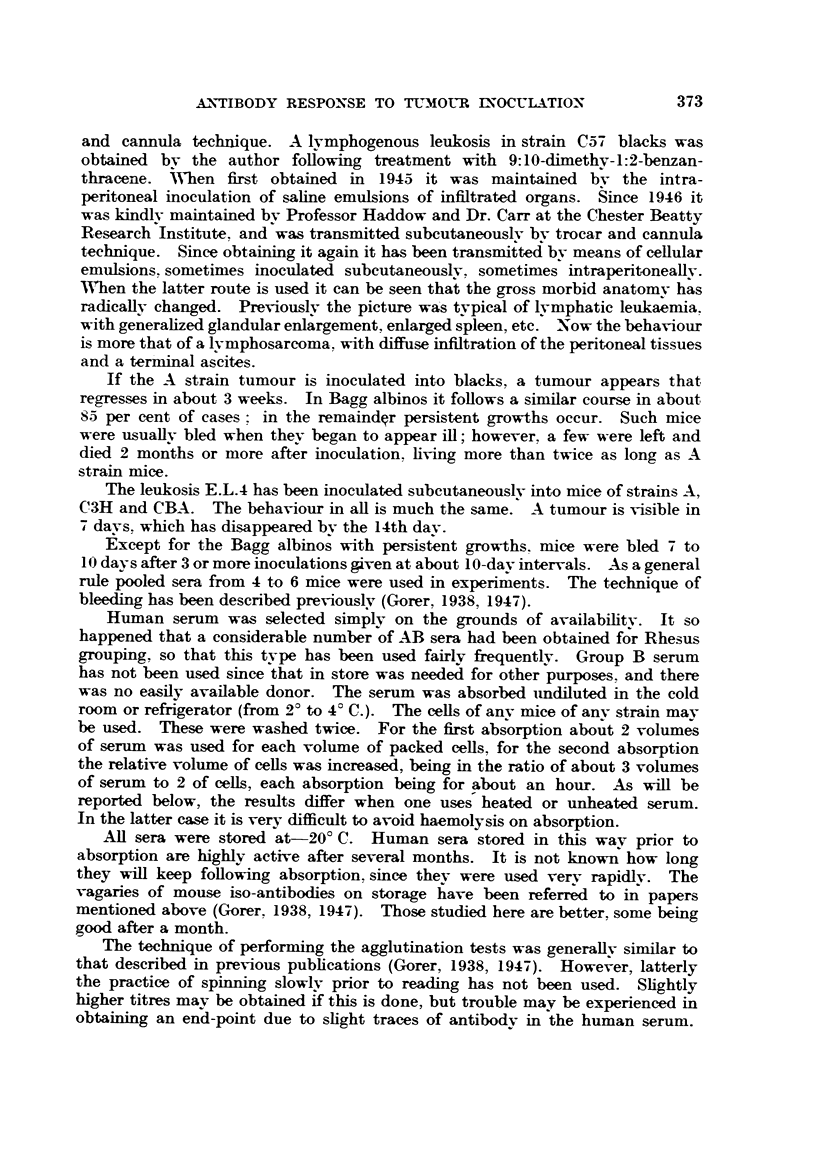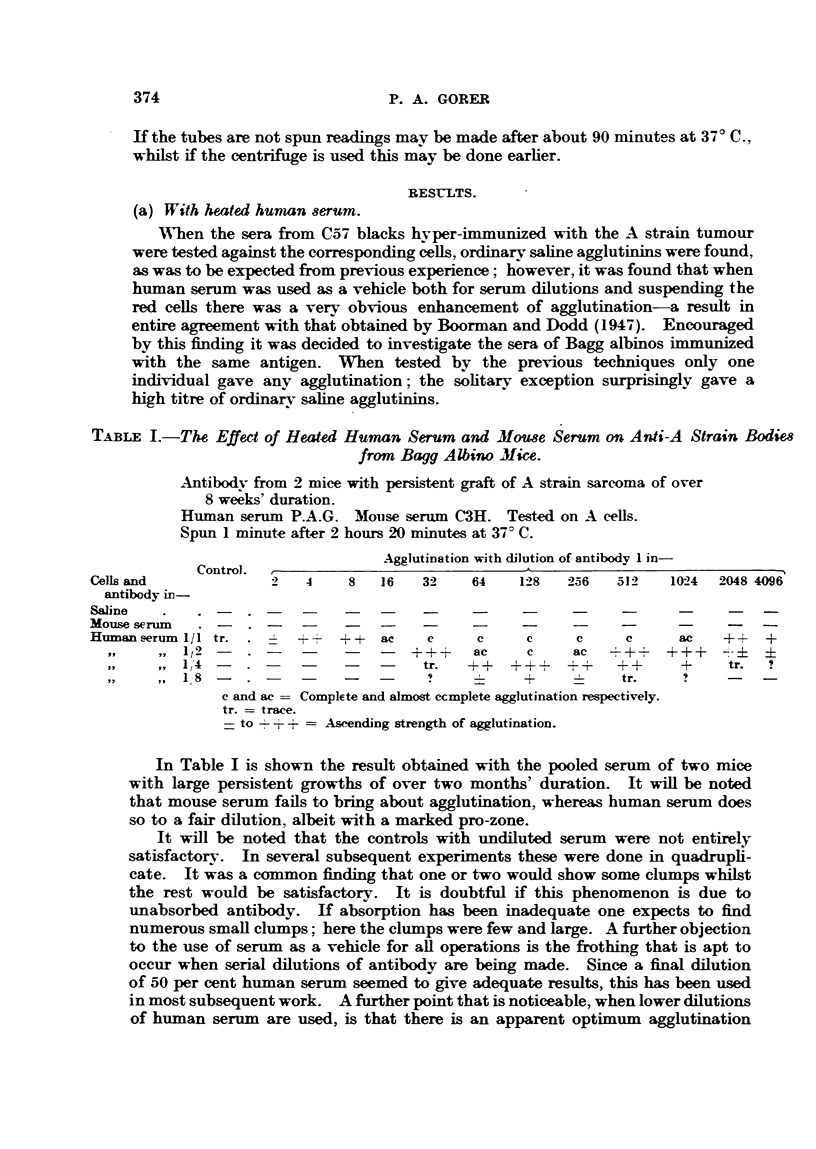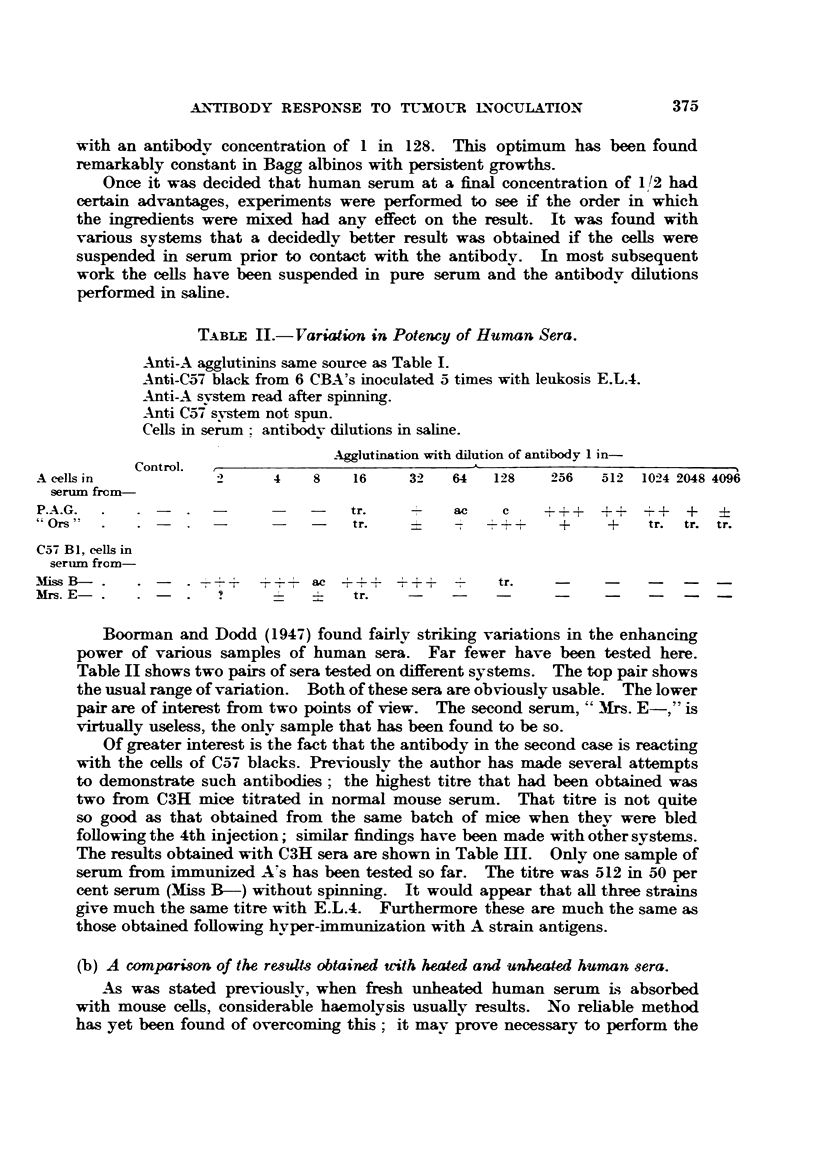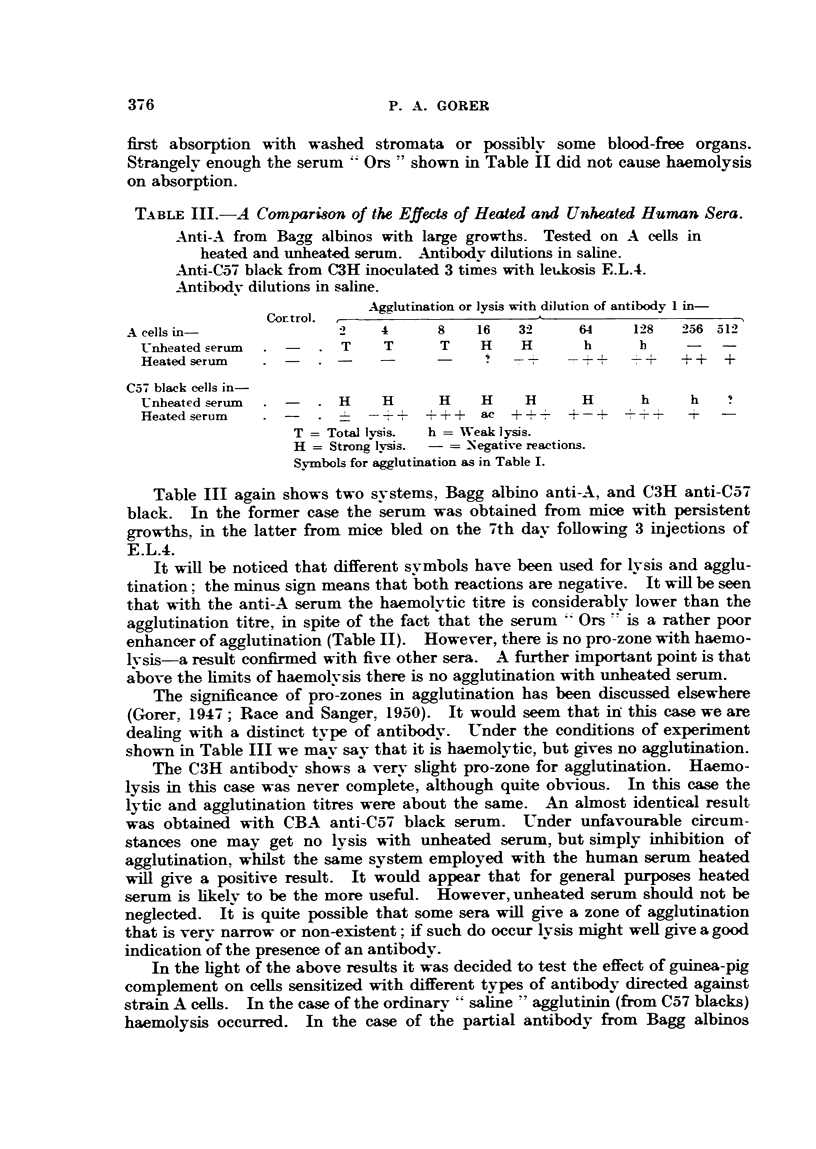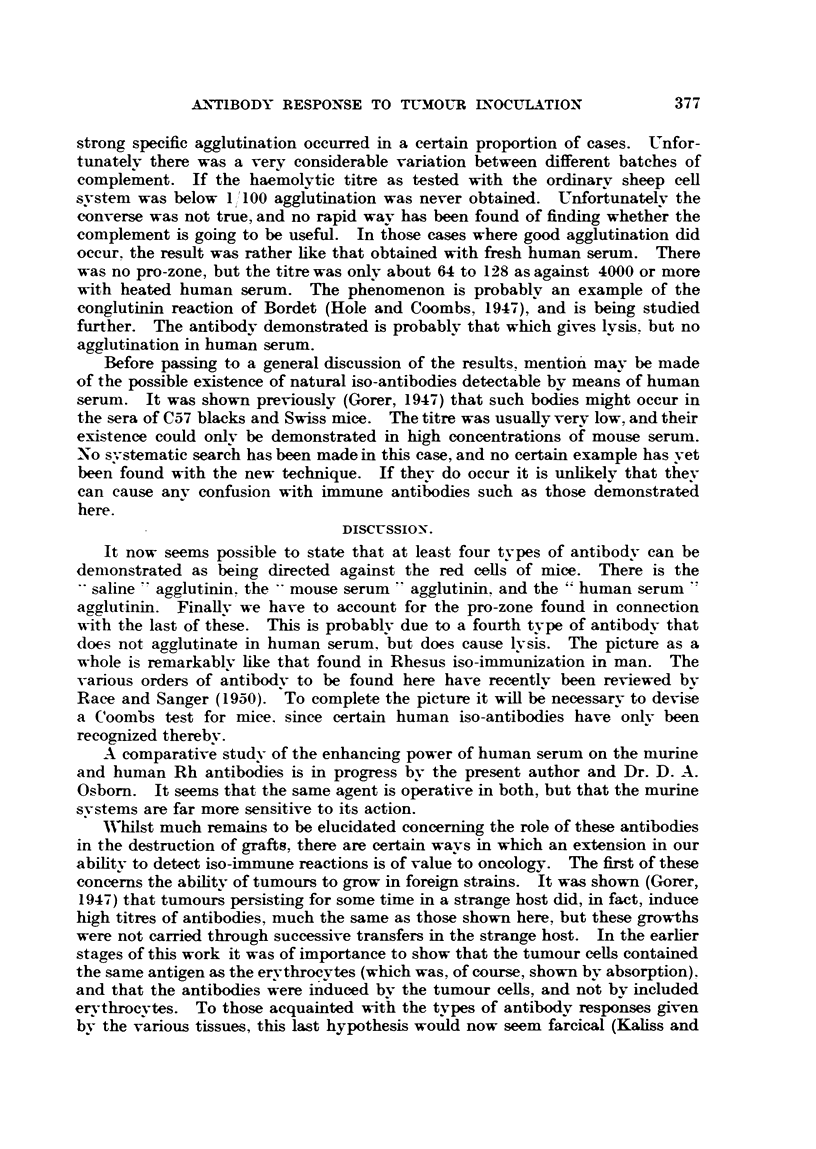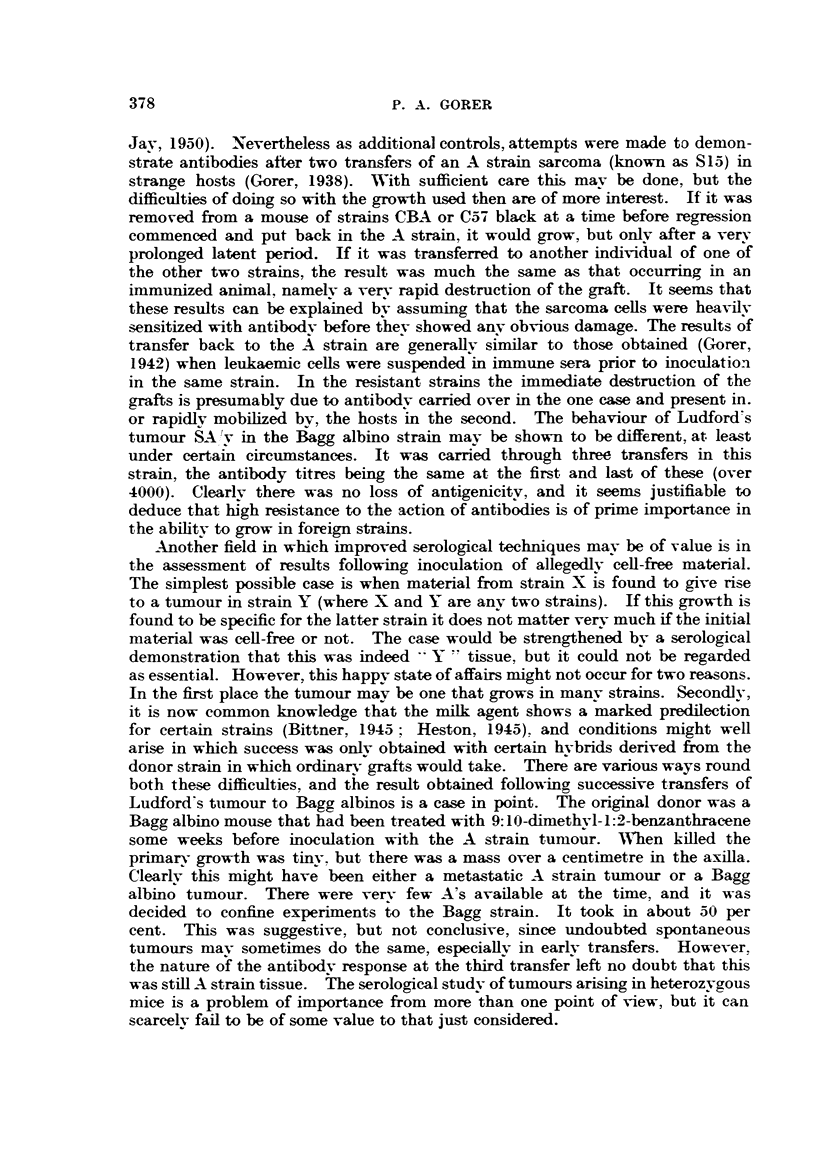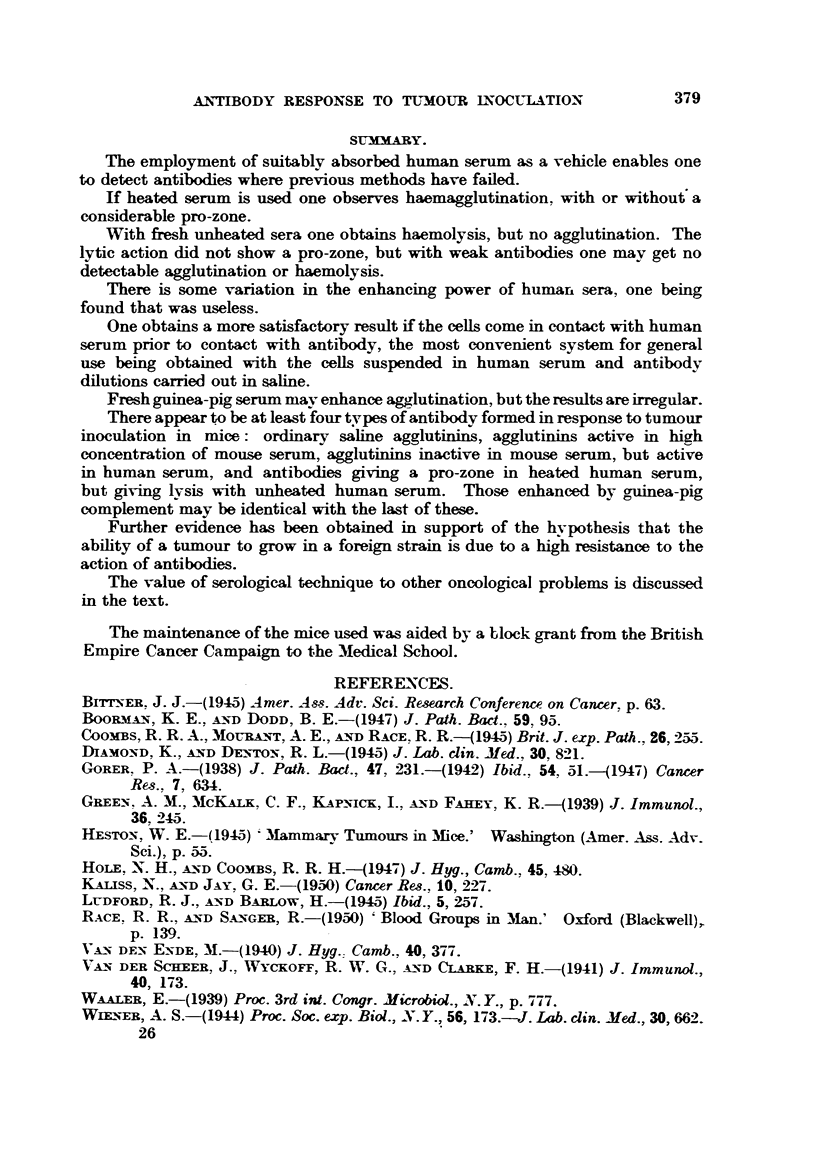# Studies in Antibody Response of Mice to Tumour Inoculation

**DOI:** 10.1038/bjc.1950.36

**Published:** 1950-12

**Authors:** P. A. Gorer


### STUDIES IN ANTIBODY RESPONSE OF MICE TO TUMOUR INOCULATION.

P. A. GORER.

*From the Department of Pathology, Guy's Hospital Medical School, London, S.E.1.*

Received for publication October 17, 1950.

It has been shown that following the inoculation of neoplastic tissues or of blood from one pure strain of mice into another, a variety of different types of antibody might be formed (Gorer, 1942, 1947). Confining our attention to the reactions obtained with the erythrocytes of the donor strain, it was found that three different types of antibody could be demonstrated: the ordinary agglutinin, an agglutinin that was active in high concentrations of normal mouse serum, and " blocking " antibodies. The last could only be demonstrated by their inhibiting action with the ordinary agglutinins. The albumin technique of Diamond and Denton (1945) was not applicable, since mouse cells are lysed by albumin. Attempts to produce a Coombs serum (Coombs, Mourant and Race, 1945) by inoculating rabbits with normal mouse plasma were not successful. This is not without interest, since it may indicate that the blocking antibody globulin of mice is absent from normal serum, or present in too low a concentration to elicit antibodies in the rabbit. Such a state of affairs is not without precedence. Thus van den Ende (1940) showed that guinea-pigs inoculated with normal rabbit serum would not give precipitation with purified anti-pneumococcal antibody. Similarly physico-chemical studies in horse sera show the presence of a new type of molecule following immunization (Green, McKalk, Kapnick and Fahey, 1939; van der Scheer, Wyckoff and Clarke, 1941). Whilst these questions will have to be cleared up, it was decided to attempt other methods first.

It was thought that some animal sera might enhance agglutination, and a few experiments were performed whilst the author was at the Roscoe Jackson Laboratory with rabbit and horse sera. In both these cases it was obvious that the absorption of natural anti-mouse agglutinins from the undiluted sera would be a formidable task. The work of Waaler (1939), Wiener (1944, 1945) and Boorman and Dodd (1947) suggested the value of human serum in enhancing agglutination, and the results obtained thereby are reported below.

### Materials and methods.

The mice used were of well-known strains, Strong A, C57 black, Bagg Albino (Strain C), C3H and CBA. Methods of bleeding, etc., have been given in the papers quoted (Gorer, 1938, 1947). A sarcoma arising in the stroma of an A strain mammary cancer known as S/Ay was obtained through the kindness of Dr. Ludford (Ludford and Barlow, 1945), and maintained by the ordinary trocar and cannula technique. A lymphogenous leukosis in strain C57 blacks was obtained by the author following treatment with 9:10-dimethy-1:2-benzanthracene. When first obtained in 1945 it was maintained by the intraperitoneal inoculation of saline emulsions of infiltrated organs. Since 1946 it was kindly maintained by Professor Haddow and Dr. Carr at the Chester Beatty Research Institute, and was transmitted subcutaneously by trocar and cannula technique. Since obtaining it again it has been transmitted by means of cellular emulsions, sometimes inoculated subcutaneously, sometimes intraperitoneally. When the latter route is used it can be seen that the gross morbid anatomy has radically changed. Previously the picture was typical of lymphatic leukaemia, with generalized glandular enlargement, enlarged spleen, etc. Now the behaviour is more that of a lymphosarcoma, with diffuse infiltration of the peritoneal tissues and a terminal ascites.

If the A strain tumour is inoculated into blacks, a tumour appears that regresses in about 3 weeks. In Bagg albinos it follows a similar course in about 85 per cent of cases ; in the remainder persistent growths occur. Such mice were usually bled when they began to appear ill; however, a few were left and died 2 months or more after inoculation, living more than twice as long as A strain mice.

The leukosis E.L.4 has been inoculated subcutaneously into mice of strains A, C3H and CBA. The behaviour in all is much the same. A tumour is visible in 7 days, which has disappeared by the 14th day.

Except for the Bagg albinos with persistent growths, mice were bled 7 to 10 days after 3 or more inoculations given at about 10-day intervals. As a general rule pooled sera from 4 to 6 mice were used in experiments. The technique of bleeding has been described previously (Gorer, 1938, 1947).

Human serum was selected simply on the grounds of availability. It so happened that a considerable number of AB sera had been obtained for Rhesus grouping, so that this type has been used fairly frequently. Group B serum has not been used since that in store was needed for other purposes, and there was no easily available donor. The serum was absorbed undiluted in the cold room or refrigerator (from 2° to 4° C.). The cells of any mice of any strain may be used. These were washed twice. For the first absorption about 2 volumes of serum was used for each volume of packed cells, for the second absorption the relative volume of cells was increased, being in the ratio of about 3 volumes of serum to 2 of cells, each absorption being for about an hour. As will be reported below, the results differ when one uses heated or unheated serum. In the latter case it is very difficult to avoid haemolysis on absorption.

All sera were stored at—20° C. Human sera stored in this way prior to absorption are highly active after several months. It is not known how long they will keep following absorption, since they were used very rapidly. The vagaries of mouse iso-antibodies on storage have been referred to in papers mentioned above (Gorer, 1938, 1947). Those studied here are better, some being good after a month.

The technique of performing the agglutination tests was generally similar to that described in previous publications (Gorer, 1938, 1947). However, latterly the practice of spinning slowly prior to reading has not been used. Slightly higher titres may be obtained if this is done, but trouble may be experienced in obtaining an end-point due to slight traces of antibody in the human serum.

If the tubes are not spun readings may be made after about 90 minutes at 37° C., whilst if the centrifuge is used this may be done earlier.

### RESULTS.

(a) *With heated human serum.*

When the sera from C57 blacks hyper-immunized with the A strain tumour were tested against the corresponding cells, ordinary saline agglutinins were found, as was to be expected from previous experience; however, it was found that when human serum was used as a vehicle both for serum dilutions and suspending the red cells there was a very obvious enhancement of agglutination—a result in entire agreement with that obtained by Boorman and Dodd (1947). Encouraged by this finding it was decided to investigate the sera of Bagg albinos immunized with the same antigen. When tested by the previous techniques only one individual gave any agglutination; the solitary exception surprisingly gave a high titre of ordinary saline agglutinins.

TABLE I.—*The Effect of Heated Human Serum and Mouse Serum on Anti-A Strain Bodies from Bagg Albino Mice.*

Antibody from 2 mice with persistent graft of A strain sarcoma of over 8 weeks' duration.
Human serum P.A.G. Mouse serum C3H. Tested on A cells.
Spun 1 minute after 2 hours 20 minutes at 37° C.

| Cells and antibody in— | Control. | Agglutination with dilution of antibody 1 in— 2 | 4 | 8 | 16 | 32 | 64 | 128 | 256 | 512 | 1024 | 2048 | 4096 |
|---|---|---|---|---|---|---|---|---|---|---|---|---|---|
| Saline | — | — | — | — | — | — | — | — | — | — | — | — | — |
| Mouse serum | — | — | — | — | — | — | — | — | — | — | — | — | — |
| Human serum 1/1 | tr. | ± | ++ | ++ | ac | c | c | c | c | c | ac | ++ | + |
| ,, ,, 1/2 | — | — | — | — | — | +++ | ac | c | ac | +++ | +++ | +± | ± |
| ,, ,, 1/4 | — | — | — | — | — | tr. | ++ | +++ | ++ | ++ | + | tr. | ? |
| ,, ,, 1/8 | — | — | — | — | — | ? | ± | + | ± | tr. | ? | — | — |

c and ac = Complete and almost complete agglutination respectively.

tr. = trace.

± to +++ = Ascending strength of agglutination.

In Table I is shown the result obtained with the pooled serum of two mice with large persistent growths of over two months' duration. It will be noted that mouse serum fails to bring about agglutination, whereas human serum does so to a fair dilution, albeit with a marked pro-zone.

It will be noted that the controls with undiluted serum were not entirely satisfactory. In several subsequent experiments these were done in quadruplicate. It was a common finding that one or two would show some clumps whilst the rest would be satisfactory. It is doubtful if this phenomenon is due to unabsorbed antibody. If absorption has been inadequate one expects to find numerous small clumps; here the clumps were few and large. A further objection to the use of serum as a vehicle for all operations is the frothing that is apt to occur when serial dilutions of antibody are being made. Since a final dilution of 50 per cent human serum seemed to give adequate results, this has been used in most subsequent work. A further point that is noticeable, when lower dilutions of human serum are used, is that there is an apparent optimum agglutination with an antibody concentration of 1 in 128. This optimum has been found remarkably constant in Bagg albinos with persistent growths.

Once it was decided that human serum at a final concentration of 1/2 had certain advantages, experiments were performed to see if the order in which the ingredients were mixed had any effect on the result. It was found with various systems that a decidedly better result was obtained if the cells were suspended in serum prior to contact with the antibody. In most subsequent work the cells have been suspended in pure serum and the antibody dilutions performed in saline.

TABLE II.—*Variation in Potency of Human Sera.*

Anti-A agglutinins same source as Table I.
Anti-C57 black from 6 CBA's inoculated 5 times with leukosis E.L.4.
Anti-A system read after spinning.
Anti C57 system not spun.
Cells in serum ; antibody dilutions in saline.

| | | Agglutination with dilution of antibody 1 in— | | | | | | | | | | | |
|---|---|---|---|---|---|---|---|---|---|---|---|---|---|
| A cells in serum from— | Control. | 2 | 4 | 8 | 16 | 32 | 64 | 128 | 256 | 512 | 1024 | 2048 | 4096 |
| P.A.G. | — | — | — | — | tr. | + | ac | c | +++ | ++ | ++ | + | ± |
| " Ors " | — | — | — | — | tr. | ± | + | +++ | + | + | tr. | tr. | tr. |
| C57 B1, cells in serum from— | | | | | | | | | | | | | |
| Miss B— | — | +++ | +++ | ac | +++ | +++ | + | tr. | — | — | — | — | — |
| Mrs. E— | — | ? | ± | ± | tr. | — | — | — | — | — | — | — | — |

Boorman and Dodd (1947) found fairly striking variations in the enhancing power of various samples of human sera. Far fewer have been tested here. Table II shows two pairs of sera tested on different systems. The top pair shows the usual range of variation. Both of these sera are obviously usable. The lower pair are of interest from two points of view. The second serum, " Mrs. E—," is virtually useless, the only sample that has been found to be so.

Of greater interest is the fact that the antibody in the second case is reacting with the cells of C57 blacks. Previously the author has made several attempts to demonstrate such antibodies ; the highest titre that had been obtained was two from C3H mice titrated in normal mouse serum. That titre is not quite so good as that obtained from the same batch of mice when they were bled following the 4th injection; similar findings have been made with other systems. The results obtained with C3H sera are shown in Table III. Only one sample of serum from immunized A's has been tested so far. The titre was 512 in 50 per cent serum (Miss B—) without spinning. It would appear that all three strains give much the same titre with E.L.4. Furthermore these are much the same as those obtained following hyper-immunization with A strain antigens.

(b) *A comparison of the results obtained with heated and unheated human sera.*

As was stated previously, when fresh unheated human serum is absorbed with mouse cells, considerable haemolysis usually results. No reliable method has yet been found of overcoming this ; it may prove necessary to perform the first absorption with washed stromata or possibly some blood-free organs. Strangely enough the serum " Ors " shown in Table II did not cause haemolysis on absorption.

TABLE III.—*A Comparison of the Effects of Heated and Unheated Human Sera.*

Anti-A from Bagg albinos with large growths. Tested on A cells in heated and unheated serum. Antibody dilutions in saline.
Anti-C57 black from C3H inoculated 3 times with leukosis E.L.4. Antibody dilutions in saline.

| | Control. | Agglutination or lysis with dilution of antibody 1 in— | | | | | | | | |
|---|---|---|---|---|---|---|---|---|---|---|
| A cells in— | | 2 | 4 | 8 | 16 | 32 | 64 | 128 | 256 | 512 |
| Unheated serum | — | T | T | T | H | H | h | h | — | — |
| Heated serum | — | — | — | — | ? | ++ | +++ | ++ | ++ | + |
| C57 black cells in— | | | | | | | | | | |
| Unheated serum | — | H | H | H | H | H | H | h | h | ? |
| Heated serum | — | ± | +++ | +++ | ac | +++ | +++ | +++ | + | — |

T = Total lysis. h = Weak lysis.
H = Strong lysis. — = Negative reactions.
Symbols for agglutination as in Table I.

Table III again shows two systems, Bagg albino anti-A, and C3H anti-C57 black. In the former case the serum was obtained from mice with persistent growths, in the latter from mice bled on the 7th day following 3 injections of E.L.4.

It will be noticed that different symbols have been used for lysis and agglutination; the minus sign means that both reactions are negative. It will be seen that with the anti-A serum the haemolytic titre is considerably lower than the agglutination titre, in spite of the fact that the serum " Ors " is a rather poor enhancer of agglutination (Table II). However, there is no pro-zone with haemolysis—a result confirmed with five other sera. A further important point is that above the limits of haemolysis there is no agglutination with unheated serum.

The significance of pro-zones in agglutination has been discussed elsewhere (Gorer, 1947; Race and Sanger, 1950). It would seem that in this case we are dealing with a distinct type of antibody. Under the conditions of experiment shown in Table III we may say that it is haemolytic, but gives no agglutination.

The C3H antibody shows a very slight pro-zone for agglutination. Haemolysis in this case was never complete, although quite obvious. In this case the lytic and agglutination titres were about the same. An almost identical result was obtained with CBA anti-C57 black serum. Under unfavourable circumstances one may get no lysis with unheated serum, but simply inhibition of agglutination, whilst the same system employed with the human serum heated will give a positive result. It would appear that for general purposes heated serum is likely to be the more useful. However, unheated serum should not be neglected. It is quite possible that some sera will give a zone of agglutination that is very narrow or non-existent; if such do occur lysis might well give a good indication of the presence of an antibody.

In the light of the above results it was decided to test the effect of guinea-pig complement on cells sensitized with different types of antibody directed against strain A cells. In the case of the ordinary " saline " agglutinin (from C57 blacks) haemolysis occurred. In the case of the partial antibody from Bagg albinos strong specific agglutination occurred in a certain proportion of cases. Unfortunately there was a very considerable variation between different batches of complement. If the haemolytic titre as tested with the ordinary sheep cell system was below 1/100 agglutination was never obtained. Unfortunately the converse was not true, and no rapid way has been found of finding whether the complement is going to be useful. In those cases where good agglutination did occur, the result was rather like that obtained with fresh human serum. There was no pro-zone, but the titre was only about 64 to 128 as against 4000 or more with heated human serum. The phenomenon is probably an example of the conglutinin reaction of Bordet (Hole and Coombs, 1947), and is being studied further. The antibody demonstrated is probably that which gives lysis, but no agglutination in human serum.

Before passing to a general discussion of the results, mention may be made of the possible existence of natural iso-antibodies detectable by means of human serum. It was shown previously (Gorer, 1947) that such bodies might occur in the sera of C57 blacks and Swiss mice. The titre was usually very low, and their existence could only be demonstrated in high concentrations of mouse serum. No systematic search has been made in this case, and no certain example has yet been found with the new technique. If they do occur it is unlikely that they can cause any confusion with immune antibodies such as those demonstrated here.

### DISCUSSION.

It now seems possible to state that at least four types of antibody can be demonstrated as being directed against the red cells of mice. There is the "saline" agglutinin, the "mouse serum" agglutinin, and the "human serum" agglutinin. Finally we have to account for the pro-zone found in connection with the last of these. This is probably due to a fourth type of antibody that does not agglutinate in human serum, but does cause lysis. The picture as a whole is remarkably like that found in Rhesus iso-immunization in man. The various orders of antibody to be found here have recently been reviewed by Race and Sanger (1950). To complete the picture it will be necessary to devise a Coombs test for mice, since certain human iso-antibodies have only been recognized thereby.

A comparative study of the enhancing power of human serum on the murine and human Rh antibodies is in progress by the present author and Dr. D. A. Osborn. It seems that the same agent is operative in both, but that the murine systems are far more sensitive to its action.

Whilst much remains to be elucidated concerning the role of these antibodies in the destruction of grafts, there are certain ways in which an extension in our ability to detect iso-immune reactions is of value to oncology. The first of these concerns the ability of tumours to grow in foreign strains. It was shown (Gorer, 1947) that tumours persisting for some time in a strange host did, in fact, induce high titres of antibodies, much the same as those shown here, but these growths were not carried through successive transfers in the strange host. In the earlier stages of this work it was of importance to show that the tumour cells contained the same antigen as the erythrocytes (which was, of course, shown by absorption), and that the antibodies were induced by the tumour cells, and not by included erythrocytes. To those acquainted with the types of antibody responses given by the various tissues, this last hypothesis would now seem farcical (Kaliss and

Jay, 1950). Nevertheless as additional controls, attempts were made to demonstrate antibodies after two transfers of an A strain sarcoma (known as S15) in strange hosts (Gorer, 1938). With sufficient care this may be done, but the difficulties of doing so with the growth used then are of more interest. If it was removed from a mouse of strains CBA or C57 black at a time before regression commenced and put back in the A strain, it would grow, but only after a very prolonged latent period. If it was transferred to another individual of one of the other two strains, the result was much the same as that occurring in an immunized animal, namely a very rapid destruction of the graft. It seems that these results can be explained by assuming that the sarcoma cells were heavily sensitized with antibody before they showed any obvious damage. The results of transfer back to the A strain are generally similar to those obtained (Gorer, 1942) when leukaemic cells were suspended in immune sera prior to inoculation in the same strain. In the resistant strains the immediate destruction of the grafts is presumably due to antibody carried over in the one case and present in. or rapidly mobilized by, the hosts in the second. The behaviour of Ludford's tumour SA/y in the Bagg albino strain may be shown to be different, at least under certain circumstances. It was carried through three transfers in this strain, the antibody titres being the same at the first and last of these (over 4000). Clearly there was no loss of antigenicity, and it seems justifiable to deduce that high resistance to the action of antibodies is of prime importance in the ability to grow in foreign strains.

Another field in which improved serological techniques may be of value is in the assessment of results following inoculation of allegedly cell-free material. The simplest possible case is when material from strain X is found to give rise to a tumour in strain Y (where X and Y are any two strains). If this growth is found to be specific for the latter strain it does not matter very much if the initial material was cell-free or not. The case would be strengthened by a serological demonstration that this was indeed "Y" tissue, but it could not be regarded as essential. However, this happy state of affairs might not occur for two reasons. In the first place the tumour may be one that grows in many strains. Secondly, it is now common knowledge that the milk agent shows a marked predilection for certain strains (Bittner, 1945; Heston, 1945), and conditions might well arise in which success was only obtained with certain hybrids derived from the donor strain in which ordinary grafts would take. There are various ways round both these difficulties, and the result obtained following successive transfers of Ludford's tumour to Bagg albinos is a case in point. The original donor was a Bagg albino mouse that had been treated with 9:10-dimethyl-1:2-benzanthracene some weeks before inoculation with the A strain tumour. When killed the primary growth was tiny, but there was a mass over a centimetre in the axilla. Clearly this might have been either a metastatic A strain tumour or a Bagg albino tumour. There were very few A's available at the time, and it was decided to confine experiments to the Bagg strain. It took in about 50 per cent. This was suggestive, but not conclusive, since undoubted spontaneous tumours may sometimes do the same, especially in early transfers. However, the nature of the antibody response at the third transfer left no doubt that this was still A strain tissue. The serological study of tumours arising in heterozygous mice is a problem of importance from more than one point of view, but it can scarcely fail to be of some value to that just considered.

SUMMARY.

The employment of suitably absorbed human serum as a vehicle enables one to detect antibodies where previous methods have failed.

If heated serum is used one observes haemagglutination, with or without a considerable pro-zone.

With fresh unheated sera one obtains haemolysis, but no agglutination. The lytic action did not show a pro-zone, but with weak antibodies one may get no detectable agglutination or haemolysis.

There is some variation in the enhancing power of human sera, one being found that was useless.

One obtains a more satisfactory result if the cells come in contact with human serum prior to contact with antibody, the most convenient system for general use being obtained with the cells suspended in human serum and antibody dilutions carried out in saline.

Fresh guinea-pig serum may enhance agglutination, but the results are irregular.

There appear to be at least four types of antibody formed in response to tumour inoculation in mice: ordinary saline agglutinins, agglutinins active in high concentration of mouse serum, agglutinins inactive in mouse serum, but active in human serum, and antibodies giving a pro-zone in heated human serum, but giving lysis with unheated human serum. Those enhanced by guinea-pig complement may be identical with the last of these.

Further evidence has been obtained in support of the hypothesis that the ability of a tumour to grow in a foreign strain is due to a high resistance to the action of antibodies.

The value of serological technique to other oncological problems is discussed in the text.

The maintenance of the mice used was aided by a block grant from the British Empire Cancer Campaign to the Medical School.

REFERENCES.

Bittner, J. J.—(1945) *Amer. Ass. Adv. Sci. Research Conference on Cancer*, p. 63.

Boorman, K. E., and Dodd, B. E.—(1947) *J. Path. Bact.*, **59**, 95.

Coombs, R. R. A., Mourant, A. E., and Race, R. R.—(1945) *Brit. J. exp. Path.*, **26**, 255.

Diamond, K., and Denton, R. L.—(1945) *J. Lab. clin. Med.*, **30**, 821.

Gorer, P. A.—(1938) *J. Path. Bact.*, **47**, 231.—(1942) *Ibid.*, **54**, 51.—(1947) *Cancer Res.*, **7**, 634.

Green, A. M., McKalk, C. F., Kapnick, I., and Fahey, K. R.—(1939) *J. Immunol.*, **36**, 245.

Heston, W. E.—(1945) 'Mammary Tumours in Mice.' Washington (Amer. Ass. Adv. Sci.), p. 55.

Hole, N. H., and Coombs, R. R. H.—(1947) *J. Hyg., Camb.*, **45**, 480.

Kaliss, N., and Jay, G. E.—(1950) *Cancer Res.*, **10**, 227.

Ludford, R. J., and Barlow, H.—(1945) *Ibid.*, **5**, 257.

Race, R. R., and Sanger, R.—(1950) 'Blood Groups in Man.' Oxford (Blackwell), p. 139.

Van den Ende, M.—(1940) *J. Hyg., Camb.*, **40**, 377.

Van der Scheer, J., Wyckoff, R. W. G., and Clarke, F. H.—(1941) *J. Immunol.*, **40**, 173.

Waaler, E.—(1939) *Proc. 3rd int. Congr. Microbiol., N.Y.*, p. 777.

Wiener, A. S.—(1944) *Proc. Soc. exp. Biol., N.Y.*, **56**, 173.—*J. Lab. clin. Med.*, **30**, 662.